# Bone Marrow Derived Mesenchymal Stem Cells Inhibit Inflammation and Preserve Vascular Endothelial Integrity in the Lungs after Hemorrhagic Shock

**DOI:** 10.1371/journal.pone.0025171

**Published:** 2011-09-28

**Authors:** Shibani Pati, Michael H. Gerber, Tyler D. Menge, Kathryn A. Wataha, Yuhai Zhao, John Adam Baumgartner, Jing Zhao, Phillip A. Letourneau, Maria P. Huby, Lisa A. Baer, John R. Salsbury, Rosemary A. Kozar, Charles E. Wade, Peter A. Walker, Pramod K. Dash, Charles S. Cox, Marie-Francoise Doursout, John B. Holcomb

**Affiliations:** 1 Department of Surgery and Center for Translational Injury Research, University of Texas Health Science Center at Houston, Houston, Texas, United States of America; 2 Department of Neurobiology and Anatomy, University of Texas Health Science Center at Houston, Houston, Texas, United States of America; 3 Department of Pediatric Surgery, University of Texas Health Science Center at Houston, Houston, Texas, United States of America; 4 Department of Anesthesiology, University of Texas Health Science Center at Houston, Houston, Texas, United States of America; Heart Center Munich, Germany

## Abstract

Hemorrhagic shock (HS) and trauma is currently the leading cause of death in young adults worldwide. Morbidity and mortality after HS and trauma is often the result of multi-organ failure such as acute lung injury (ALI) and acute respiratory distress syndrome (ARDS), conditions with few therapeutic options. Bone marrow derived mesenchymal stem cells (MSCs) are a multipotent stem cell population that has shown therapeutic promise in numerous pre-clinical and clinical models of disease. In this paper, *in vitro* studies with pulmonary endothelial cells (PECs) reveal that conditioned media (CM) from MSCs and MSC-PEC co-cultures inhibits PEC permeability by preserving adherens junctions (VE-cadherin and β-catenin). Leukocyte adhesion and adhesion molecule expression (VCAM-1 and ICAM-1) are inhibited in PECs treated with CM from MSC-PEC co-cultures. Further support for the modulatory effects of MSCs on pulmonary endothelial function and inflammation is demonstrated in our *in vivo* studies on HS in the rat. In a rat “fixed volume” model of mild HS, we show that MSCs administered IV potently inhibit systemic levels of inflammatory cytokines and chemokines in the serum of treated animals. *In vivo* MSCs also inhibit pulmonary endothelial permeability and lung edema with concurrent preservation of the vascular endothelial barrier proteins: VE-cadherin, Claudin-1, and Occludin-1. Leukocyte infiltrates (CD68 and MPO positive cells) are also decreased in lungs with MSC treatment. Taken together, these data suggest that MSCs, acting directly and through soluble factors, are potent stabilizers of the vascular endothelium and inflammation. These data are the first to demonstrate the therapeutic potential of MSCs in HS and have implications for the potential use of MSCs as a cellular therapy in HS-induced lung injury.

## Introduction

Traumatic injury is currently one of the leading causes of death worldwide. One of the hallmarks of hemorrhagic shock (HS), a condition resulting from rapid blood loss after traumatic injury, is the onset of a systemic response that results in endothelial injury, inflammation, aberrant coagulation, tissue edema and end organ injury [1]. HS is associated with a high incidence of acute lung injury (ALI) and acute respiratory distress syndrome (ARDS) (14–20% of ICU patients) that results in significant morbidity and mortality [2]–[7]. There are currently few treatments beyond supportive therapy to treat lung edema and injury. Over the past ten years, the therapeutic potential of the multipotent bone marrow derived stem cell population known as mesenchymal stem cells (MSCs) have been investigated in multiple disease states including sepsis [8], acute renal failure [9], graft vs. host disease [10], ALI [11], and myocardial infarction [12]. MSCs have multiple characteristics that make them attractive candidates for clinical translation: 1) They are relatively easy to isolate, 2) They grow rapidly in culture, and 3) They are able to home to sites of active tissue injury where they are thought to modulate inflammation and vascular function [13]. There are currently 154 clinical trials registered with Clinicaltrials.gov investigating the use of adult MSCs in a number of conditions; however, there are no clinical trials investigating the use of MSCs in HS, ALI or ARDS despite the significant pre-clinical data to support their use and therapeutic benefits. In our past work, we have shown that intravenously (IV) administered MSCs have potent stabilizing effects on the vascular endothelium in injury [14] and are capable of inhibiting blood brain barrier (BBB) permeability after traumatic brain injury (TBI) via modulation of the adherens junctions (AJs) proteins: VE-cadherin and β-catenin. In endothelial cells (ECs) these AJ proteins form molecular “zippers” between neighboring ECs, hence regulating paracellular permeability and tissue edema [15]. In addition, our past work has demonstrated that the influence of MSCs on endothelial permeability and barrier function is likely mediated by a secreted factor(s) that is released as a result of MSC-endothelial cell interactions. Based upon our past work and that of others showing that MSCs have potent anti-inflammatory effects in multiple disease states [13] we hypothesized that MSCs would have similar stabilizing effects in the lungs exposed to HS. To address our hypothesis we chose to study pulmonary endothelial cell-MSC interactions *in vitro* and the effects of MSCs *in vivo* in a rat model of HS and resuscitation.

## Methods

### Ethics Statement

All experiments involving the use of animals were in compliance with the National Institutes of Health *Guide for the Care and Use of Laboratory Animals* and were approved by the University of Texas Health Science Center at Houston's Institutional Animal Care and Use Committee, #HSC-AWC-10-073.

### Primary cells and cell lines

First passage human MSCs and PECs were purchased from Lonza (Walkersville, MD). MSCs were cultured in MSC growth media (MSC-GM, Lonza); PECs were maintained in EGM-2MV media. MSCs were used at passage 3–7 for all experiments. U937s were obtained from ATCC (Bethesda, MD) and passaged in RPMI with 10% fetal bovine serum. All cell lines were maintained at 37°C and 5% CO_2_.

### Flow cytometry

MSCs were characterized by flow cytometry as described previously [16]. PECs were treated with CM from MSCs, PEC+MSCs, and PECs alone. Cells were stimulated with TNFα, 50 ng/ml for four hours. After treatment, cells were harvested and stained with antibodies to VCAM-1 (Becton Dickinson, Franklin Lakes, NJ), ICAM-1 (Becton Dickinson), E-Selectin (Becton Dickinson), and P-Selectin (Becton Dickinson). Cells were stained with secondary antibodies and ran on a flow cytometer (BD FacsCalibur, BD Biosciences, San Jose, CA).

### Flow Cytometry of Adhesion Marker Expression on PECs

Conditioned media was made in three groups: (1) PECs alone, (2) MSCs alone, (3) PECs/MSCs co-culture. EGM-2 (Lonza) was added to wells as culture medium. After 48 hours of culture, the media was removed and stored at −80°C. PECs grown to confluence on 6 well plates and growth media was replaced with the conditioned media in the above groups. The experiment was performed in replicates of four. After 12 hour incubation, TNFα (50 ng/mL) was added to the wells and incubated for 4 hours. The cells were then collected and stained with fluorophore conjugated antibodies to I-CAM, V-CAM, E-selectin, and P-selectin (BD Biosciences). The cells were then analyzed on the BD LSR II (BD Biosciences) flow cytometer.

### Conditioned media (CM)

**Figure 1:**
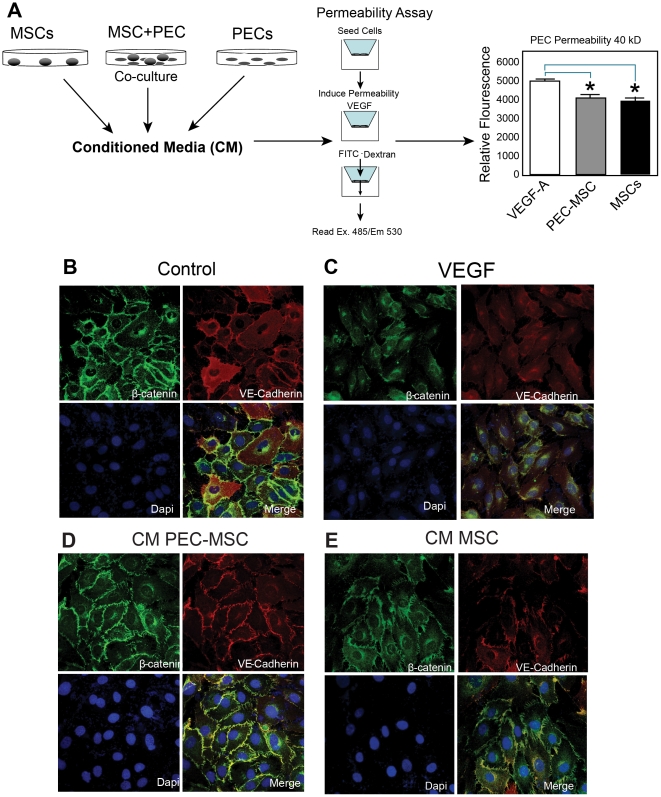
Conditioned media (CM) inhibits permeability and restores adherens junctions (AJs). Figure 1A, CM from MSCs and MSC-PEC co-cultures inhibits PEC permeability *in vitro*. A schematic depicting how CM is prepared is shown. MSCs and PECs are cultured separately and together in co-culture. CM is collected 24 hours later and used in a transwell assay of PEC permeability to 40 kD FITC-Dextran. Figure 1A shows that CM from both MSCs and MSC-PEC co-cultures inhibits permeability induced by VEGF-A (10 ng/ml). (*) signs indicate significance p<0.05 between MSC and VEGF and MSC-PEC and VEGF groups. Figures 1B–E, CM from MSCs and MSC-PEC co-cultures restore AJs. PECs were cultured overnight. After 24 hours, breakdown of AJs (β-catenin and VE-Cadherin) is induced by treatment with VEGF-A (10 ng/ml). Cells were subsequently treated with CM. Figures 1B–E show that MSC and MSC-PEC CM restored AJ breakdown as determined by confocal microscopy imaging of cultures stained with antibodies to VE-Cadherin (red) and β-Catenin (green).

PECs were co-cultured in contact with MSCs. 5×10^5^ cells were seeded on a 4.67 cm^2^ surface area. The ratio of MSCs to PECs was 0.2 determined by previously reported experiments [14], [16]. CM was harvested from co-cultures after 24 hours. See schematic in Figure 1.

### Endothelial cell permeability

Collagen coated 0.4 µm pore size inserts were obtained from BD Biosciences. PECs were seeded at 40,000 cells per insert well in a total volume of 500 µl of media and cultured for 8 hours to allow for cell attachment and adhesion. PEC monolayer permeability was tested 8 hours later by adding 10 µl of 10 mg/ml 40 kD FITC-Dextran (Sigma-Aldrich, St. Louis, MO) to the upper chamber of each well. Monolayers were treated with 10 ng/ml VEGF-A165 from R&D (Minneapolis, MN), one hour prior to addition of FITC-Dextran-40 kD. Measurements were determined with a fluorimeter using excitation and emission wavelengths of 485 nm and 530 nm, respectively, from samples obtained 30 minutes after addition of FITC-Dextran. Data represent the mean ± standard error of the mean (SEM) from three independent experiments. For experiments in which the effects of CM from MSCs or MSC/PEC co-cultures were studied, the media from PEC monolayer co-cultures that had been plated on collagen coated inserts and allowed to adhere was replaced with undiluted CM and maintained in culture for four hours prior to addition of FITC-Dextran. Data represent the mean ± SEM from three independent experiments.

### *In vitro* VE-cadherin/β-catenin staining (PECs)

Passage 3 PECs were seeded into 8-well glass chambered slides and grown for 48 hours at 37°C. The cells were then treated with basal growth media or CM from PEC-MSC co-cultures, PECs alone, or MSCs alone for 60 minutes at 37°C. To induce permeability, recombinant human VEGF-A (R&D Systems #293-VE-010, Minneapolis, MN) was added at 50 ng/mL and incubated for 30 minutes at 37°C. The cells were then fixed in 2% formaldehyde and blocked in TBST+2.5% normal goat serum for 60 minutes at room temperature. A rabbit α-VE-cadherin antibody (1∶400 dilution, Cell Signaling #2500 Beverly, MA) and a mouse α-β-catenin antibody (1∶200 dilution, Cell Signaling #2677) were applied overnight at 4°C and detected using an Alexa 488 α-rabbit antibody (1∶500 dilution, Molecular Probes #A-11034, Invitrogen, Carlsbad, CA) and an Alexa 568 α-mouse antibody (1∶500 dilution, Invitrogen Molecular Probes #A-11031, Invitrogen). The next day the slides were mounted using ProLong Gold anti-fade reagent with DAPI (Molecular Probes P-36931, Invitrogen) to obtain nuclear staining. Images of the cells were taken at 40× on a Nikon A1R confocal microscope (Nikon Instruments, Inc, Melville, NY).

### Leukocyte binding assay

PECs were grown to confluence on 96 well plates. 1×10∧4 cells/well were seeded and incubated at 37°C for 2 days or until confluent. Adhesion molecule expression was stimulated by the addition of TNFα (50 ng/ml). Cells were treated with CM for 1.5 hours. CM was generated from co-cultured cells and cells grown alone in PEC growth media. U937 cells were labeled with Calcein-AM (Invitrogen), added to wells and allowed to adhere for one hour. Non-adherent cells were gently washed away in phosphate buffered saline (PBS) and labeled cells that remained were quantified by fluorescent reading on the Synergy 2 Mircoplate Reader (Biotek, Winooski, VT) at 490 nm wavelength excitation and 520 nm emission.

### Statistical analysis

Data in all graphs is shown as mean ± SEM. One-way ANOVA was used for statistical analysis of dose response permeability studies, adhesion markers and *in vivo* quantitation of cytokines and inflammatory infiltrates.

### Animals and HS model

*Surgical Preparation*: Under isoflurane anesthesia, male Sprague-Dawley rats (Harlan Laboratories, Indianapolis, IN) were instrumented by placement of 23 g sterile polyurethane tubing (Access Technologies, Skokie, IL.) into the femoral artery and vein. The catheters were subcutaneously tunneled and exited the dorsal neck. After a 3-day recovery period, the arterial catheter was connected to a physiological pressure transducer (MLT844, distributed by AD Instruments, Colorado Springs, CO). Pressures and heart rates were recorded by a PowerLab 4/30 (AD Instruments). Rats were randomly assigned to one of four groups. **Group I:** Sham. Animals received no injury or treatment. **Group II:** HS. Animals received hemorrhage alone. **Group III:** HS+resuscitation with Lactated Ringer's Solution (LR). Animals received hemorrhage and were then treated with LR (3× shed blood volume). **Group IV:** LR+MSC. Animals experienced hemorrhage, were resuscitated with LR as Group III and also 2×10^6^ MSCs were administered with LR at 1 and 24 hours, re-suspended in 100 µl of PBS. Blood samples were taken at 2 hours post hemorrhage and pressures recorded at baseline, end of hemorrhage, 1, 2, and 96 hours after hemorrhage. Hemorrhage was achieved by withdrawing 2 ml/100 g body weight of blood from the venous or arterial catheter over 10 minutes. This yielded a 30% blood loss. This procedure was the same for all animals receiving hemorrhage. Resuscitation was administered at one hour post hemorrhage. LR was administered at a volume of 3 times the shed blood volume over 30 minutes. After 96 hours, the rats were euthanized under isoflurane anesthesia.

### Tissue harvesting and sectioning protocol for lung tissue

The lungs were harvested, and used for measurements of wet-to-dry ratios, molecular biology studies (left side, one lobe) histopathology, and immunohistochemistry (right side, four lobes). Ninety-six hours after the protocol was started, animals were euthanized under intubated anesthesia (5% isoflurane through a 16×2″G Terumo Surflo I.V catheter). The chest was opened and a hemostatic clamp was placed on the left main bronchus and 2.5 ml of a solution of 0.5 M sucrose and OCT (Tissue Tek embedding medium for frozen tissue, Sakura Fineteck USA Inc., Torrance, CA) were mixed in equal volume and injected through the endotracheal tube, to fill the right lobe. The right main bronchus was clamped, cut over the clamp to secure the embedding media inside the lobes and tied with 4-O silk. The tissue was immediately dropped in HistoPrep disposable base molds (Fisher Scientific, Pittsburg, PA) filled with OCT, wrapped in labeled foil and snap frozen in liquid nitrogen. This side of the lung was stored at −80°C and is cryosectioned later for immunohistochemistry staining. Another piece of lung was fixed in formalin for paraffin fixation and histopathological analysis. The tissue was moved from the −80°C freezer to the cryostat (Shandon Cryotome SME, Thermo Fisher Scientific, Waltham, MA). Sections (6 µm) were cut at a working temperature of −17°C, and mounted to microscope slides (VWR Vista Vision™ HistoBond® slides, VWR International, Radnor, PA). The slides were kept at −80°C until stained.

### Immunohistochemistry

Lung sections (6 µm) were cut on a cryostat, mounted on slides, post-fixed for 20 minutes with −20°C methanol, and rehydrated in PBS. Sections were incubated in primary antibodies (1.0 µg/ml in PBS containing 2.5% goat serum and 0.25% Triton X-100) for 48 hours at 4°C, washed in PBS, and then incubated with Alexa Fluor-conjugated species-specific secondary antibodies for 3 hours at room temperature. Sections were cover slipped with Fluormount-G (Fisher Scientific), and imaged using an upright Olympus BX-51 microscope (Bio-Rad Laboratories, Hercules, CA) attached to a MagnaFire digital camera (Optronics, Goleta, CA). Bisbenzamide H was added in the final TBS wash sequence to obtain nuclear staining. Antibodies used: monoclonal to α-myeloperoxidase antibody (MPO) (Abcam, Cambridge, MA), CD68 antibody (Abcam), VE-cadherin (Cell Signaling), Occludin-1 (Invitrogen), Claudin-1 (Invitrogen) and Human mitochondrial antibody (Millipore, Concord, MA).

### Lung wet-to-dry ratios

The clamp was removed from the left bronchus, and the left lung was harvested. One piece of this lobe was taken, moved to a small microcentrifuge tube, snap frozen in liquid nitrogen and stored at −80°C for molecular biology studies (measurement of cytokines in tissue). The other piece was placed on a labeled plastic dish. The dish containing the lung was weighed immediately after harvesting (day 0 weight), and then stored in a 50°C oven to dry for 72 hours and reweighed (day 3 weight) to calculate the wet-to-dry ratio. The ratios between the day 0 and day 3 weights are the wet-dry/dry ratios and are considered a measurement of lung edema.

### Cytokine and chemokines by Bioplex (tissue and serum)

Cytokines were measured in the tissue at four days and in the serum at two hours. Two hours was picked as the main time point to determine inflammatory changes since previous studies with this model had shown that the maximum changes in inflammatory cytokines occur between 1–4 hours after HS [17]–[19]. The Bio-Plex cytokine assay system (Bio-Rad Laboratories) and concentrations of IL-1α, IL-1β, IL-6, IL-10, MIP-1a, MCP-1 and TNFα were simultaneously evaluated using a commercially available multiplex bead-based immunoassay (Rat 9-Plex, Bio-Rad Laboratories). The assay was performed per the manufacturer's instructions and the details have been previously published by our group and others [20]. High standard curves (low RP1 target value) for each soluble cytokine were used, ranging from 2 to 32,000 pg/ml. A minimum of 100 beads per cytokine region were evaluated and recorded. Values with a coefficient of variation beyond 10% were not included in the final data analysis. All samples were run in duplicate.

## Results

### Conditioned media from MSCs inhibits pulmonary endothelial cell permeability *in vitro*

In our past work with human umbilical vein endothelial cells (HUVECS), we have shown HUVECs in contact with MSCs produce a soluble factor(s) that inhibits paracellular permeability and may be responsible for the enhanced barrier integrity in MSC/HUVEC co-cultures [14]. To gain further insight into the mechanism by which MSCs could potentially modulate PEC permeability, we investigated whether MSCs could inhibit paracellular permeability in PECs. We tested the CM from cells grown in two different formats (MSCs alone and PEC+MSCs) for their ability to reduce paracellular permeability induced by VEGF-A. In each format, the total number of cells seeded in the culture was held constant at 2×10^5^ cells per well. For the co-cultured conditions, MSCs were added to give a final MSC to PEC ratio of 0.2; we have previously used this ratio to demonstrate effects on endothelial function [14], [16]. Conditioned media was harvested 24 hours after seeding and tested for their effect on permeability (Figure 1A). Permeability to 40 kD FITC-Dextran for each culture condition was assessed and normalized to the permeability values obtained using CM from PECs alone. Figure 1B demonstrates that the permeability of cultured PECs is induced by VEGF-A and is inhibited by CM from MSC as well as MSC-PEC. This finding differs from our previous results with HUVECs, where the effects on permeability were only noted in the CM from HUVEC-MSC co-cultures, indicating that the effects may be dependent on the endothelial cell type since the endothelium is highly heterogeneous and varies greatly between the organ of origin [21].

### MSCs increase VE-cadherin/β-catenin interaction at the cell surface of PECs

As mentioned previously, AJs (VE-cadherin and β-catenin) are critical regulators of vascular integrity and permeability [22], [23]. To determine if β-catenin is involved in the decrease in barrier permeability we observed (Figure 1B), we looked at the effects of the MSCs on β-catenin and VE-cadherin localization in PECs treated with CM from MSCs and MSCs+PECs as described above. Figures 1C–E reveal that CM from MSCs and MSC-PEC co-cultures restore AJs (β-catenin and VE-cadherin) staining at the surface of PECs that have been treated with VEGF-A. PECs treated with CM from MSCs and MSC-PEC co-cultures demonstrate enhanced VE-cadherin and β-catenin immunoreactivity at the membrane and an enhanced propensity to grow in clusters. Furthermore our findings with CM suggest, as we have found in past work, that a secreted factor(s) from MSCs is responsible for improving the barrier integrity of PECs. Interestingly, in these studies with PECs, we find that CM from MSCs alone has potent effects on AJs and permeability.

### MSCs inhibit leukocyte adhesion to PECs

**Figure 2:**
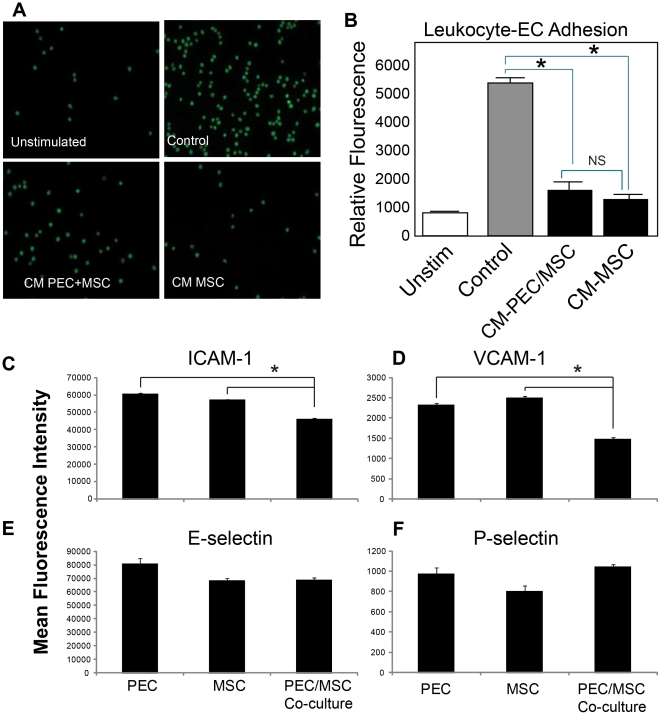
CM inhibits adhesion of U937s and adhesion molecule expression in PECs. Figures 2A and 2B, CM from MSC and MSC+PEC co-cultures inhibits adhesion of U937 to PECs. Calcein labeled U937 cells were allowed to adhere to PECs that had been pre-treated with CM from PEC and MSC-PEC co-cultures. Cultures were stimulated with TNFα and U937s were added to the wells. After gentle washing, bound cells were quantitatively and qualitatively assessed. Figure 2A shows decreased binding of U937 (green dots) in CM treated cultures. Figure 2B shows quantitative significant decreases in CM treated cultures as indicated by (*), which signifies p<0.05 between control (TNFα treated cells) and MSC CM and control and CM-CO (MSC-PEC CM). “Unstim” represents the group not treated with TNFα. Eight wells were included in each group (n = 8). Figures 2C–F, CM from MSC+PEC co-cultures inhibits PECs expression of adhesion molecules VCAM-1 and ICAM-1. Conditioned media was made in three groups: (1) PECs alone, (2) MSCs alone, (3) PECs/MSCs co-culture. EGM-2 (Lonza) was added to wells as culture medium. After 48 hours of culture the media was removed and stored at −80°C. PECs grown to confluence on 6 well plates and growth media was replaced with the conditioned media in the above groups. The experiment was performed in replicates of four. After 12 hour incubation, TNFα (50 ng/mL) was added to the wells and incubated for 4 hours. The cells were then collected and stained with fluorophore conjugated antibodies to I-CAM, V-CAM, E-selectin, and P-selectin. The cells treated with PEC/MSC conditioned media demonstrated decreased I-CAM and V-CAM expression compared to both those treated with PEC or MSC conditioned media(p<0.01, ANOVA). This difference was not seen in E-selectin or P-selectin.

Multiple factors are responsible for the compromise of the endothelial barrier in HS-induced lung injury. In addition to factors such as hypoxia and thrombin, inflammatory changes caused by cytokine and chemokine release, leukocyte adhesion, diapedesis and infiltration into the lungs have all been shown to contribute to the clinical severity and outcome in ALI/ARDS [4]. We sought to determine if MSCs modulate leukocyte binding to PECs stimulated with the inflammatory cytokine TNFα. In these studies, PECs were treated with CM from MSCs and MSC-PEC co-cultures. To stimulate inflammatory cell (U937) binding, PECs were treated with TNFα (50 ng/ml) which increased U937 cell binding as depicted in Figure 2A. U937 cells are a monocytoid line that we have previously used to study leukocyte-endothelial adhesion [24]. Binding of calcein-labeled cells to treated PECs was quantified by relative fluorimetric. Binding studies reveal that pre-treatment of the PECs with CM media from MSC or MSC-PEC co-cultures inhibit U937 binding to the PECs (Figure 2B). Flow cytometric analysis of the treated PECs reveals that CM from MSC+PECs co-culture, but not MSC CM, inhibit TNFα mediated increases in ICAM-1, and VCAM-1, suggesting a contact-mediated effect. E-selectin and P-selectin were unchanged by either treatment, suggesting that the effects of the MSC-PEC CM is on firm adhesion of leukocytes and not tethering and rolling processes typically regulated by the selectins (Figures 2C–2F). Taken together, our findings suggest that the therapeutic effects of MSCs are not solely due to enhancement of barrier integrity, but do have direct effects on inflammatory cell binding to the endothelium. It is of interest to note that we find effects of the MSC CM media alone on EC permeability, but in the case of leukocyte binding, the effects are limited to co-cultured MSC-PEC CM. These findings suggest that the soluble factors mediating barrier integrity may be different from those mediating inflammation and leukocyte adhesion, indicating a decoupling of these effects. To determine if our *in vitro* findings translate into similar effects in a rat model of HS, we administered MSCs IV after hemorrhage with specific focus upon the effects on endothelial integrity and inflammation in the lungs.

### IV Administration of MSCs in rats subjected to HS does not affect mean arterial pressure (MAP) in treated animals

**Figure 3:**
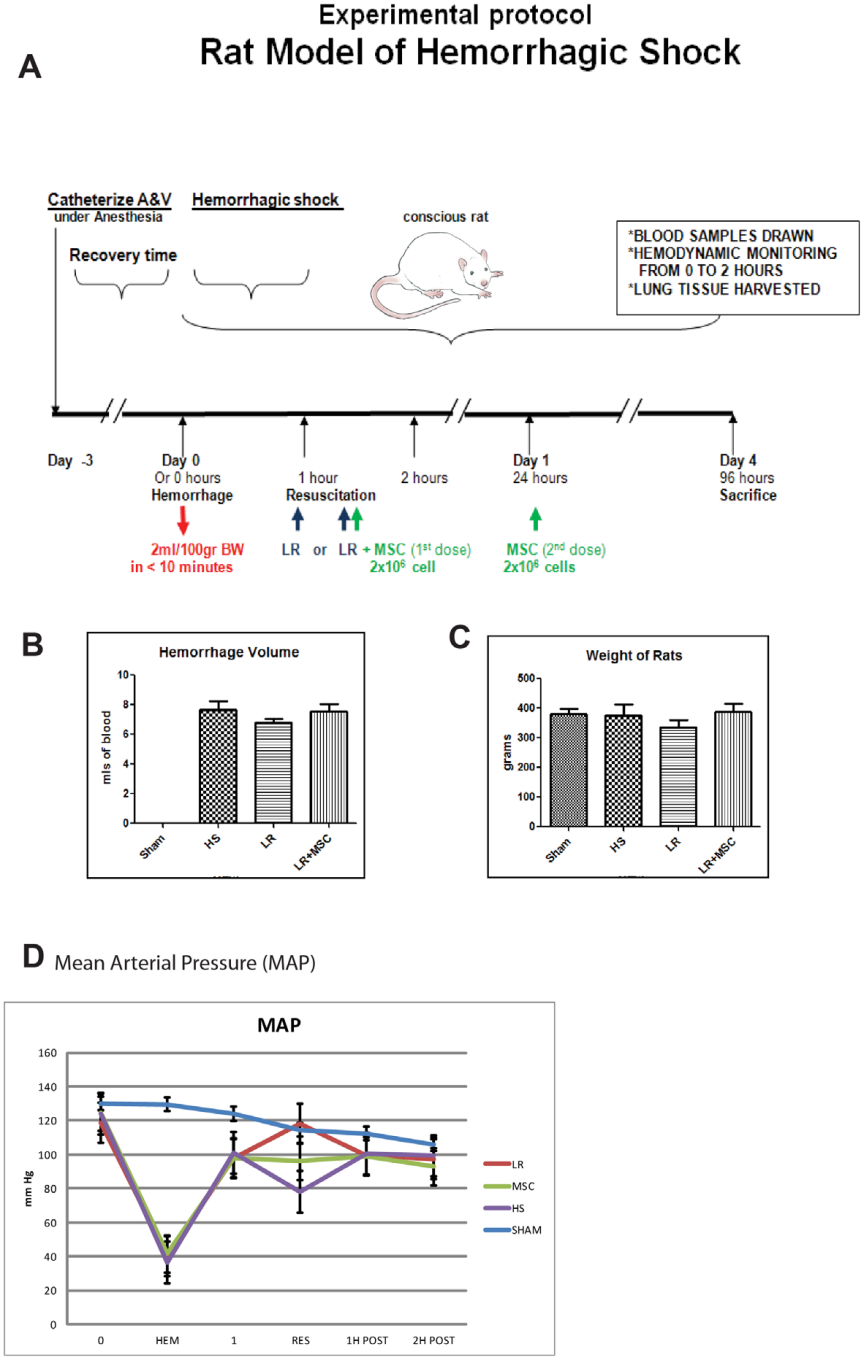
*In Vivo* IV MSCs do not alter mean arterial pressure (MAP) in rat model of hemorrhagic shock and resuscitation. Figure 3A shows a schematic of the *in vivo* rat model. Animals were pre-instrumented three days prior to hemorrhage. Hemorrhage of fixed volume at a rate of 2 ml/100 g/10 minutes was performed. One hour later, resuscitation of Lactated Ringer's (LR) (3× shed blood) was administered. MSCs at a dose of 2×10^6^ were administered with LR at 1 and 24 hours post hemorrhage. Blood was drawn at 0, 2 and 96 hours. Tissues were harvested on day four. Figures 3B and 3C show that hemorrhage volume and weight of rats is similar in all groups. Figure 3D shows that MAP drops respectively in all hemorrhaged groups and returns to normal by 2 hours post-hemorrhage. MSC administration does not affect MAP.

Using a well characterized rat model of mild, non lethal HS [14], [25], we sought to determine if administration of IV MSCs after hemorrhage affected MAP. Rats were randomly assigned to four main groups with anticipated 100% survival in all groups. Animals underwent hemorrhage with removal of 2 ml/100 g of blood over 10 minutes. MAP fell to approximately 30 mmHg in all groups. After a 60 minute period of shock, animals were resuscitated. Groups were as follows (Figure 3A): **Group I:** Sham- animals received no injury or treatment. **Group II:** HS- animals received hemorrhage alone. **Group III:** HS+LR. Animals underwent hemorrhage and were then resuscitated with LR. **Group IV:** LR+MSC- rats were resuscitated with LR as in Group III plus 2×10^6^ MSCs were administered with LR at 1 hour post hemorrhage and a second dose in 100 µl of LR at 24 hours after hemorrhage. Dosing of MSCs was determined by our past work and that of others in disease models of vascular stability [16], [26]. The logic behind our selection of LR was to mimic the early stages of resuscitation in trauma patients when MSCs could potentially be used as an adjunct to resuscitation. The goal would be to administer MSCs at an early time point to promote vascular stability and prevent the development of ALI/ARDS or multi-organ failure (MOF). Human MSCs were obtained commercially (see methods) from four young, healthy donors for use in all experiments. Our choice to use human MSCs in the rat was prompted by our past work showing no acute evidence of rejection and previous findings that human MSCs may act differently then rat MSCs [27], [28]. Prior to using these cells for *in vitro* and *in vivo* experiments, fluorescence activated cell sorting (FACS) analysis was performed to examine the expression of characteristic MSC cell surface markers. Cells were confirmed to display characteristic MSC cell surface markers [16], including high levels of CD44 and CD105, and an absence of CD31 expression (data not shown). Figures 3B and 3C show that there are no significant changes in hemorrhage volume and weight respectively between animals in all three groups subjected to hemorrhage. Figure 3D demonstrates no differences in MAP in all three groups. The MAP dropped soon after hemorrhage and was essentially restored in all groups by one hour post hemorrhage.

### IV administration of MSCs in rats subjected to HS inhibits edema and inflammatory cell infiltrates in the lungs of treated animals

**Figure 4:**
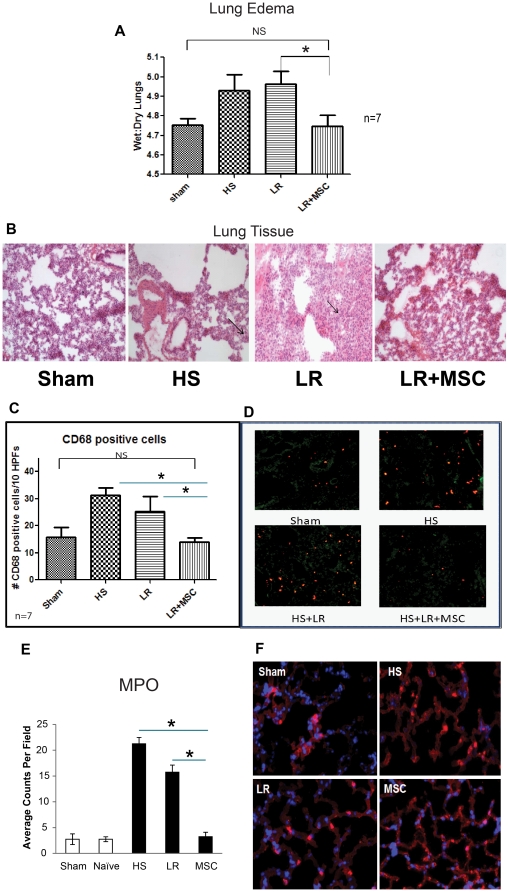
MSCs inhibit lung edema and inflammatory cells in HS rats. Figure 4A, MSCs inhibit interstitial lung edema. Wet-to-dry ratios of lung tissue calculated as described in materials and methods demonstrate that mild edema is produced by HS in rat lungs. (* indicates p<0.05 between LR and LR+MSC). This small increase in edema remains with the administration of LR, but is diminished in animals receiving LR+MSCs, thereby indicating that MSCs inhibit HS induced lung edema. Figure 4B shows H&E staining of MSC treated lungs suggested decreased edema and injury. Representative lung sections from the four groups were stained for H&E. Gross histopathological analysis demonstrates decreased edema and inflammation in MSC treated lungs. HS and HS+LR treated groups show edema and inflammatory infiltrates (see arrows). Figures 4C and 4D show that inflammatory CD68 positive cells are decreased in MSC treated lungs. Lung sections were stained for CD68, a marker of inflammatory monocytes and macrophage infiltrates. Five sections were analyzed per animal (n = 5) and 8–10 high power fields were counted for fluorescent positive (CD68+) cells. Figure 4C shows that CD68+ cells are significantly decreased in MSC treated animals (* indicates p<0.05). MSC treated animals were not significantly different from Shams. Figure 4D shows representative fluorescent images of the CD68+ cells in the lungs. Figures 4E and 4F, Myeloperoxidase positive cells are decreased in MSC treated lungs. Myeloperoxidase (MPO) staining of MSC treated lungs shows decreased MPO positive cells. Representative stained sections are shown in Figure 4F.

To determine if lung edema and injury were generated by the hemorrhagic insult, lungs were taken for wet-to-dry analysis. Wet-to-dry-analysis (Figure 4A) revealed that mild edema is present in Group II (HS alone). Resuscitation with LR (Group III) did not significantly increase the amount of lung edema above HS (Group II), but MSCs (Group IV) did indeed significantly decrease the amount of edema compared to LR (Group III) and HS (Group II), thus this data suggests that IV MSCs may have modulator effects upon vascular stability in the lungs after HS. Histopathological (H&E) analysis (Figure 4B) of tissue sections from rat lungs indicate that animals treated with MSCs show attenuated injury. Further histopathological analysis reveals that MSC treated animals have decreased numbers of CD68 positive cells, a general surface marker of leukocytes and lymphocytes, in the lungs compared to HS (Group II) and LR (Group III) animals. Figure 4D shows qualitative differences in CD68 staining between Group IV and Group II and III. Figure 4C quantitatively demonstrates decreased numbers of CD68 positive cells in MSC-treated lungs with numbers similar to sham animals (Group I). Further evidence of a decrease in neutrophilic infiltration in MSC treated lungs is evident from immunofluorescence staining for myeloperoxidase (MPO) positive cells (Figure 4E and 4F). MPO staining is significantly decreased in Group IV (MSCs) compared to Group II (HS) and Group III (LR). Once again, numbers of MPO positive cells in MSC-treated animals are close to those found in Group I (sham), consistent with inhibition of pulmonary inflammation.

### IV administration of MSCs in rats subjected to HS inhibits TNFα, MCP-1, MIP-1α and augments IL-10

**Figure 5:**
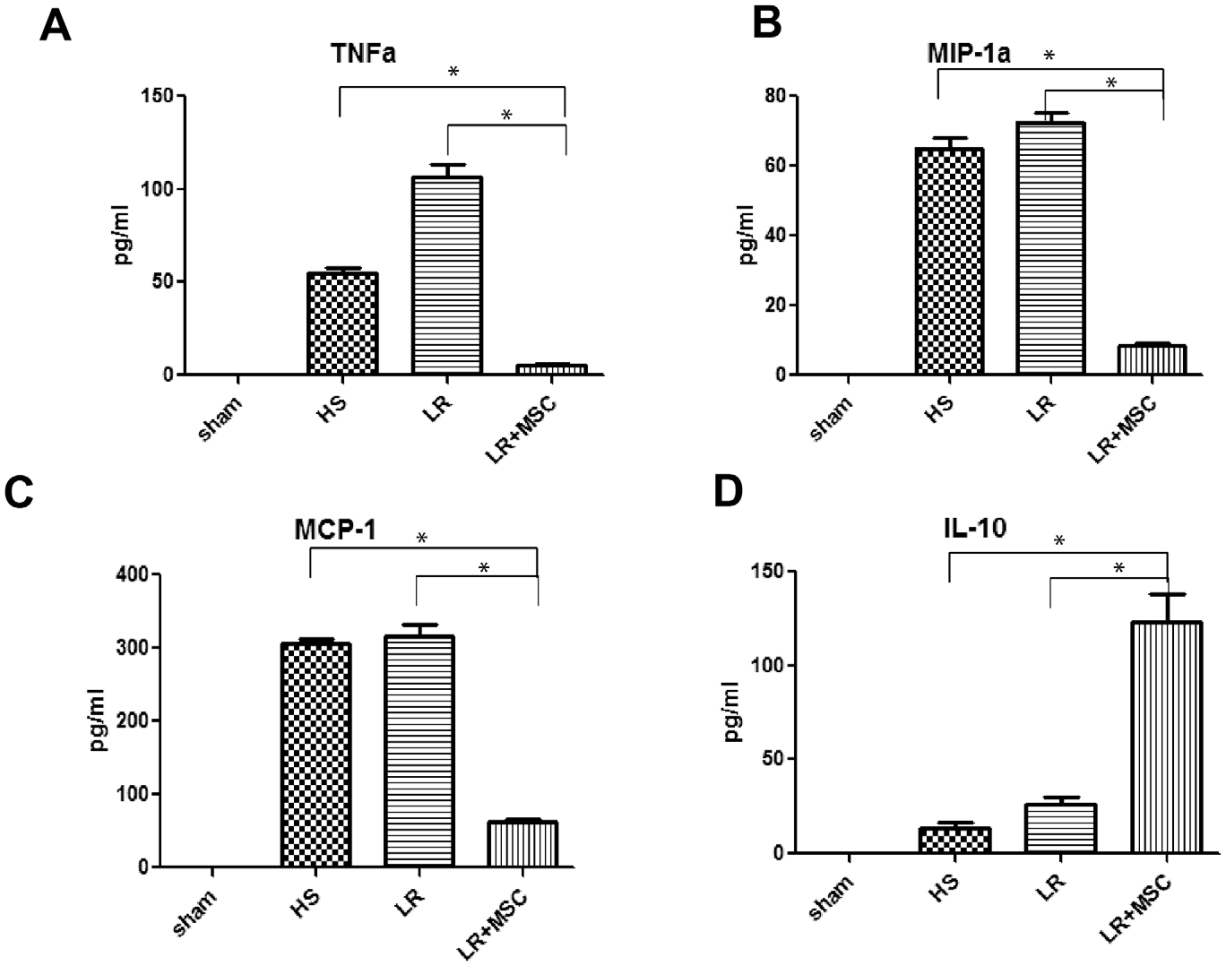
TNFα, MIP-1α, and MCP-1 are decreased and anti-inflammatory cytokine IL-10 is increased by MSC treatment. Blood was drawn at two hours post-hemorrhage and analyzed for serum levels of TNFα, MCP-1, IL-10 and MIP-1α. As shown in Figures 5A–C, bioplex analysis of serum shows that MCP-1, TNFα, and MIP-1α all significantly increase after HS and HS+LR. This rise is significantly decreased by MSC administration (* indicates p<0.05 for HS vs. LR+MSC and LR vs. LR+MSC). Figure 5D shows that anti-inflammatory cytokine IL-10 is significantly increased by MSC (Figure 5D).

In light of our findings with changes in inflammatory cells in the lungs, we sought to determine if inflammatory cytokines and chemokines changed in animals treated with MSCs. Serum collected at two hours post-hemorrhage was subjected to multiplex bead-based cytokine analysis as described in the methods. Two hours was picked as the main time point to determine inflammatory cytokine and chemokine changes since previous studies with this model had shown that the maximum changes in inflammatory cytokines occur between 1–4 hours after HS. Serum from animals subjected to HS revealed significant rises in TNFα, MCP-1, MIP-1α, and IL-10 (Figures 5A, 5B, 5C, and 5D). Animals treated with MSCs (Group IV) showed decreases in TNFα, MCP-1 and MIP-1α compared to HS (Group II) and LR (Group III). Interestingly, MSC-treated animals demonstrated a significant rise in the anti-inflammatory cytokine IL-10 (Figure 5D). Tissue levels and serum levels of these same four cytokines and chemokines were unchanged in lung tissue that was harvested at 96 hours post-hemorrhage, indicating resolution of the inflammatory process from this insult by four days post hemorrhage (data not shown).

### MSCs preserve vascular tight junctions and AJs in the lungs of MSC treated rats

**Figure 6:**
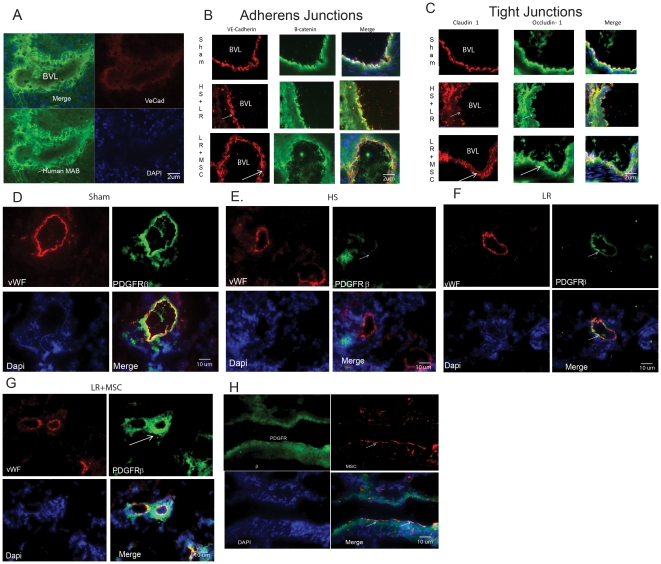
MSCs in the lung vasculature. Figures 6A-C show, IV MSCs localize to the lung vasculature and preserve integrity of AJs and tight junctions (TJs). Figure 6A, MSCs (see white arrow) were detected around blood vessels in the lungs (BVL = blood vessel lumen) using a human mitochondrial antibody (green) showing staining in lung tissue from MSC treated rats. (red = VE-Cadherin and DAPI staining nuclei -blue). Figure 6B: Staining of blood vessels in lungs (BVL = blood vessel lumen) for VE-Cadherin (red) and β-catenin (green) shows that in the LR group, HS and resuscitation compromise the continuity of the AJs (see white arrow). This continuity is preserved in MSC treated animals. Figure 6C: Similar findings are found for TJs staining of Occludin-1 (green) and Claudin-1 (red). MSCs preserve compromise of TJs in the lungs (see white arrows). Morphological changes (thickening) in AJs and TJs are noted in MSC treated lung vessels (long white arrows). Figures 6D–H show MSCs preserve PDGFRβ positive pericytes on lung microvasculature after HS. Sectioned lung tissue was stained for PDGFRβ (green) to identify pericytes or smooth muscle (SM) cells and vWF (red) to identify blood vessels. Figures 6E and 6F shows that HS and LR groups show diminished or compromised PDGFRβ staining (small white arrows), indicating decreased pericyte/SM cell coverage on microvasculature. This is increased above Sham (Figure 6D) in MSC treated animals (Figure 6G-see large white arrow). Figure 6H shows that MSCs (red) contribute to some, but not all, of the increased PDGFRβ (green) staining found in treated lungs.

Previously, we have shown that IV MSCs administered after TBI inhibit BBB permeability through preservation of AJs and tight junctions in the brain [17]. AJs and tight junctions regulate vascular integrity and paracellular permeability in vascular endothelium. VE-cadherin-VE-cadherin homophilic interactions on endothelial cells are crucial for maintaining normal vascular integrity and inhibiting permeability [15], [29]. These junctions link the extracellular environment to the intracellular environment by recruiting and binding β-catenin, which then binds to the actin cytoskeleton via other intermediate proteins within the cytoplasm [15]. Since we found a decrease in lung edema with MSCs in our rodent model of HS, we sought to determine if MSCs preserve or increase tight junctions in the lungs. To confirm presence and determine the location of the MSCs in the lungs of treated animals, we stained lung tissue for human mitochondria using an antibody that does not cross-react with murine mitochondria (see methods), thereby allowing us to locate the human MSCs. Immunoflourescent staining of the lungs reveals that the majority of the MSCs are located in the perivascular space adjacent to vessel lumens, with some present in the lung parenchyma as well (Figure 6A). Figure 6B shows that rats subjected to HS and LR resuscitation exhibit diminished staining of AJ protein VE-cadherin, which is spared significantly in MSC-treated animals. Morphological analysis reveals a slight thickening the AJs (Figure 6B). Furthermore, similar sparing effects are found for Occludin-1 and Claudin-1 (Figure 6C) in MSC treated rats. Both markers are tight junction proteins, indicating that MSCs may work in a similar fashion in HS as we had found in TBI, to reinforce and enhance vascular integrity after injury. These molecular changes are consistent with our *in vitro* findings demonstrating that MSC CM inhibits lung endothelial cells, permeability and AJ integrity.

### MSCs enhance pericyte/smooth muscle cell presence in lung capillaries

The endothelial-organ barrier is composed of multiple components acting together to maintain barrier integrity. It is heterogeneous throughout the body and its function and structure are dependent upon the organ of interest. To maintain the stable environment required for the effective function, capillaries in the lung consist of a single layer of endothelial cells interconnected by tight junction proteins, AJs and pericytes/smooth muscle cells that reside adjacent to the endothelial cells and result in a continuous basal lamina [30]–[32]. Breakdown of the barrier, which occurs in lung injury, leads to infiltration of blood components, inflammatory cells and fluid into lung alveoli and interstitial space, causing increased free radical production, inflammation, edema and compromised gas exchange and lung function. Pericytes, although decreased in number in the lungs, have been shown to be key components reinforcing barrier integrity. To study pericyte/smooth muscle cell and basal lamina structure in the lungs, lung sections were stained for PDGF Receptor β, a marker for pericytes and vascular smooth muscle cells (SMC). In small capillaries (<10 um) few SMCs are present. Qualitative immunoflourescent staining for PDGFRβ in lung microvasculature reveals positive staining (green) around capillary blood vessels, defined by vWF staining (red) (Figure 6D). Animals subjected to HS reveal diminished pericyte/SMC staining in the lungs (Figure 6E), which is preserved and seemingly enhanced in MSC treated animals (Figure 6F). LR resuscitation (Figure 6G) also partially restores pericyte/SMC presence in the lungs. Since MSCs are known to be PDGFRβ positive and work by others has shown them to have multiple characteristics similar to pericytes [33]–[37], we co-stained lung sections for MSCs using the human mitochondrial marker and PDGFRβ. Figure 6H reveals that MSCs do indeed stain positive for PDGFRβ along the vessel lumen, but not at the perivascular periphery of the vessels, thereby indicating that the seemingly enhanced presence of pericytes/SMC in MSC treated animals is not likely due to perivascular MSCs.

## Discussion

Our work demonstrates that MSCs can have stabilizing effects upon the lung vasculature after HS. Overall we demonstrate that MSCs inhibit EC barrier permeability and preserve pulmonary endothelial cell integrity by preserving AJs, tight junctions and decreasing inflammation. I*n vitro* we find that CM (from MSCs and MSCs+PECs) inhibits PEC permeability and leukocyte adhesion respectively. These findings indicate that a soluble factor(s) produced by MSC administration could potentially be the mediator of many of the noted beneficial therapeutic effects of MSCs in disease states characterized by vascular instability and inflammation. Support for the therapeutic effects of a number of MSC derived factors has been shown by others investigating their use in lung injury [38]–[41]. In our *in vivo* rat model of mild HS, we find presence of MSCs in the vasculature of the lungs associated with decreased lung interstitial edema, preservation of AJs and decreased inflammatory infiltrates. The anti-inflammatory effect of MSCs is notable and indicated by potent decreases in inflammatory cytokines and chemokines and an increase in the anti-inflammatory cytokine, IL-10. IL-10 has been shown by others to be a key mediator of MSC action in LPS models of lung injury [8]. Interestingly, we also found an increase in the pericyte/smooth muscle component of the vascular barrier, an effect that could add to strengthening of the alveolar capillary barrier.

Abnormalities in vascular permeability leading to inflammation, tissue edema and end-organ dysfunction significantly contribute to the morbidity and mortality associated with a number of human disease processes [42]. The endothelial barrier as a target for therapy is a concept that is slowly becoming recognized as one with significant potential [43]. Both HS and septic shock are characterized by abnormal vascular permeability [1], [14], which contributes to the development of multi-organ failure and is the primary cause of shock-associated ALI and ARDS [2]. Despite the clear importance of abnormal vascular permeability in a number of human disease processes, no current therapeutic in use targets vascular permeability and its consequent adverse results. Taken together, our findings suggest that MSCs, and possibly systemic factors produced by their administration, can therapeutically target vascular permeability and inflammation through local and systemic effects.

The lung is an organ that is highly susceptible to edema and endothelial permeability after traumatic injury. ALI and ARDS are common complications with the annual incidence in ICU patients as high as 20% with coincident mortality ranging from 50–70% [3]–[7]. Increased vascular permeability has been shown to persist over the course of ARDS and is related to the clinical score of injury severity and outcome [3]. The pathophysiology of this process is still unclear, but a large number of inflammatory mediators and cells contribute to its development. In the exudative phase of lung injury, alveolar capillary endothelial cells and type I pneumocytes are compromised, thereby resulting in a loss of the barrier that prevents fluid and macromolecule leak. Inflammatory cells and cytokines lead to continued endothelial injury. The component that occurs first, endothelial permeability or inflammation, is not clear and is the subject of the current study. Our findings demonstrate that IV MSCs address both components of endothelial permeability and inflammation induced by HS. This is the first study to report the therapeutic benefits of MSCs in HS. It will be of interest to determine in future studies if MSCs mitigate outcome in a more severe model of HS.

Currently there are a number of groups investigating novel cell-based therapies for sepsis-induced ALI/ARDS [40]. MSCs possess biological properties that make them an ideal cell-based therapeutic for pathologic edema associated with trauma-related conditions [44], [45]. MSCs can be readily expanded to hundreds of millions of cells, proliferate for many passages in culture, and are easily transfected, allowing for easy *ex vivo* modification with genes of interest. In lung injury, MSCs have been shown to have potent effects in pre-clinical models of ALI induced by sepsis, characterized by lung edema and inflammation [11], [46]. In many models, MSCs were able to modulate the immune system through the release of anti-inflammatory cytokines, inhibition of inflammatory cytokines and release of lipid mediators such as prostaglandin E_2_ [8]. A recent study by Matthay and colleagues revealed that MSCs produce keratinocyte growth factor (KGF), a factor that restores lung epithelial and endothelial permeability [40]. Taken together, these published findings support our current findings that MSCs have global effects on vascular permeability and stability, likely due to soluble factors that mediate these effects.

In our future work we will study the effects of MSCs in a more severe animal model of hemorrhagic shock and trauma. This more severe model will most likely produce fulminant ALI or ARDS. It is quite typical that most hemorrhagic shock models do not produce much lung edema, but rather a modest increase in lung endothelial permeability with some interstitial edema, as we have shown. However, there is extensive clinical and pre-clinical data supporting a direct correlation between lung interstitial edema and pulmonary function [47]. Taking into account that normally the distance between the pulmonary microvasculature and the alveoli forming the alveolar capillary barrier for gas exchange is small (1–2 microns), it is likely that small perturbations in this limited interstitial area would impact gas exchange. Future studies will establish the effects of MSCs on oxygenation and lung compliance. Based upon the findings of others, we anticipate this will be the case for a severe model of HS.

**Figure 7:**
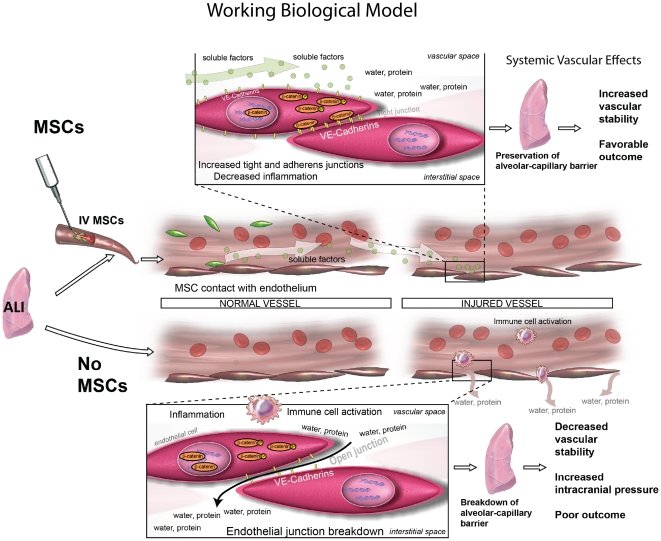
Soluble factors play a role in the effects of IV MSCs in vivo. Figure 7 shows a working biological model of how MSCs may function biologically when delivered IV after HS. MSC attach to pulmonary vascular endothelial cells in the lungs where they produce soluble factor(s) that affect vascular stability in the lungs through modulation of AJs, TJs and checking inflammation. We hypothesize that the soluble factor(s) produced promote local and systemic vascular stability.

Another future direction of this research would be to identify the soluble factor(s) that can be used as a “cell-free” therapeutic to recapitulate the beneficial effects of MSCs (Figure 7, working biological model). There are clear advantages from a translational standpoint in injured patients; MSCs administered after trauma may have potent therapeutic effects, but are difficult to harvest rapidly and administer as an autologous treatment option. Heterologous transfer is complicated by the potential for rejection and storage problems. If a soluble factor(s) can recapitulate these effects, confirmation of its presence and functional properties *in vivo*, and ultimately determination of its identity, can result in a novel “cell-free” treatment option for injured patients. Although most perceive the effect of MSCs to be due to their presence in the injured organ, many groups are realizing that secreted factors play a large role in the noted beneficial effects of stem cells in models of injury. Future pre-clinical and clinical studies in HS and trauma will assess these effects.
